# Control of the Longitudinal Compression and Transverse Focus of Ultrafast Electron Beam for Detecting the Transient Evolution of Materials

**DOI:** 10.3390/ma15020571

**Published:** 2022-01-13

**Authors:** Xintian Cai, Zhen Wang, Chaoyue Ji, Xuan Wang, Zhiyin Gan, Sheng Liu

**Affiliations:** 1 The Institute of Technological Sciences, Wuhan University, Wuhan 430072, China; caixintian@whu.edu.cn (X.C.); wang.zhen@whu.edu.cn (Z.W.); 2018106520021@whu.edu.cn (C.J.); 2 Beijing National Laboratory for Condensed Matter Physics, Institute of Physics, Chinese Academy of Sciences, Beijing 100190, China; xw@iphy.ac.cn; 3 School of Mechanical Science & Engineering, Huazhong University of Science & Technology, Wuhan 430074, China; ganzhiyin@hust.edu.cn; 4 School of Power and Mechanical Engineering, Wuhan University, Wuhan 430072, China

**Keywords:** transient evolution, detection, ultra-fast electron beam

## Abstract

Ultrafast detection is an effective method to reveal the transient evolution mechanism of materials. Compared with ultra-fast X-ray diffraction (XRD), the ultra-fast electron beam is increasingly adopted because the larger scattering cross-section is less harmful to the sample. The keV single-shot ultra-fast electron imaging system has been widely used with its compact structure and easy integration. To achieve both the single pulse imaging and the ultra-high temporal resolution, magnetic lenses are typically used for transverse focus to increase signal strength, while radio frequency (RF) cavities are generally utilized for longitudinal compression to improve temporal resolution. However, the detection signal is relatively weak due to the Coulomb force between electrons. Moreover, the effect of RF compression on the transverse focus is usually ignored. We established a particle tracking model to simulate the electron pulse propagation based on the 1-D fluid equation and the 2-D mean-field equation. Under considering the relativity effect and Coulomb force, the impact of RF compression on the transverse focus was studied by solving the fifth-order Rung–Kutta equation. The results show that the RF cavity is not only a key component of longitudinal compression but also affects the transverse focusing. While the effect of transverse focus on longitudinal duration is negligible. By adjusting the position and compression strength of the RF cavity, the beam spot radius can be reduced from 100 μm to 30 μm under the simulation conditions in this paper. When the number of single pulse electrons remains constant, the electrons density incident on the sample could be increased from $3.18\times 10^{12}$ $m^{-2}$ to $3.54\times 10^{13}$ $m^{-2}$, which is 11 times the original. The larger the electron density incident on the sample, the greater the signal intensity, which is more conducive to detecting the transient evolution of the material.

## 1. Introduction

Nowadays, people have paid more and more attention to the transient evolution of materials, such as the formation of crystal defects in the growth of wide-gap semiconductors, the nonlinear process of a femtosecond laser acting on materials, the physical phase transitions, and chemical reactions [1,2,3,4]. However, the revealing of the transient mechanism is usually limited to simulations, such as finite element, molecular dynamics, and first-principles calculations [5,6,7]. Therefore, ultra-fast detection of the materials’ transient evolution has become particularly urgent.

The commonly used methods of ultra-fast detection are ultra-fast X-ray diffraction (XRD) and ultra-fast electron [8,9]. Compared with ultra-fast XRD, ultra-fast electron detection has a larger scattering cross-section with controllable wavelength and is less harmful to the sample. First of all, X-rays have Thomson interactions with extranuclear electrons through electromagnetic fields, while electrons have long-range interactions with atomic nuclei and extranuclear electrons through Coulomb force [10,11]. The signal-to-noise ratio of ultra-fast electron detection is higher, with the scattering cross-section being 5–6 orders of magnitude higher than X-rays [12]. Secondly, with strong penetrability, X-ray detection is suitable for studying solid materials, while electron detection is suitable for studying film materials, gas molecules, and surface characteristics [13]. In addition, during the scattering process, the X-rays with the same energy can deposit about 1000 times higher energy on the sample than the electron beam, which may cause damage to the sample [14]. Moreover, the ultra-fast electronic detecting system has a much lower cost and more compact structure than the X-ray synchrotron radiation source [15]. It can even be integrated with the material growth chamber for real-time in-situ monitoring.

However, the propagation mechanism of the ultra-fast electron beam is very complicated due to the Coulomb force and the relativistic effect. Existing research shows that it is necessary to fulfill the ultra-high temporal and spatial resolution requirements to detect the materials’ transient evolution [16]. Therefore, the longitudinal and transverse dimensions of the ultra-fast electron beam must be strictly controlled. Zewail et al. were the first to study ultra-fast electron detection and reduced the temporal resolution to nearly 1 ps [17,18,19]. Luiten and Siwick et al. developed a keV single-shot ultra-fast electron detection system and designed a compact structure to reduce the temporal resolution within 100 fs [20,21]. Kochikov et al. conducted a systematic study on the relativistic model of the ultra-fast electron beam [22]. Cao and Wang et al. observed the ultra-fast process of aluminum melting and coherent phonon generation in laser-heated gold nanofilm [23,24]. Y. Qi et al. proposed a pulse-shaping self-compression method to improve the detection [25]. Pasmans et al. revealed the evolution of the longitudinal and transverse power of the electron beam under the action of the RF cavity [26]. In most works on RF-compressed UED instruments, particle tracking simulations have been performed that rigorously include the interplay between transverse and longitudinal beam properties. However, in a systemic study of the trade-offs, the longitudinal compression and the transverse focus of the electron pulse are typically analyzed as two dependent processes that do not interfere with each other [27,28,29,30]. However, with requirements for the system resolution and imaging quality of ultra-fast electron imaging continually increasing, the interaction between transverse focus and longitudinal compression should be further elucidated. For most electrons in a pulse, the resultant Coulomb force with all other electrons changes because the pulse shape changes by longitudinal compression. The changes will inevitably affect the pulse’s transverse focusing, which mainly consists of the focus radius and its position. However, the longitudinal compression and transverse focusing of the ultra-fast electron beam are usually analyzed separately. There is still a lack of research on the interaction. Moreover, there are still difficulties in revealing the evolution of the beam spot size in the time and position domain adequately by experiments.

Based on the above research, the 1-D fluid and the 2-D mean-field models were adopted to analyze the relationship between the longitudinal duration and the transverse radius. Simultaneously, the fifth-order Runge–Kutta equation was utilized for solving the ultra-fast propagation process. The effects of longitudinal compression on transverse focus and the effects of transverse focus on longitudinal duration are studied. In addition, the impact of the RF cavity on the transverse focus under different intensities and positions was simulated, which provides a theoretical basis for the extraction of weak signals in the detection of the transient evolution mechanism of materials.

## 2. Simulation Conditions and Methods

### 2.1. 1-D Fluid Model and 2-D Mean-Field Model

Since electrons are excited from the photocathode, they have a particular energy dispersion, typically 0.1–0.5 eV [25]. When the electrons reach the anode, after being accelerated by the high negative voltage, the incomplete accordant of size and direction of each electron causes the longitudinal and transverse expansion of the pulse, which is manifested as an increase in pulse duration and radius. In addition, for high energy above 30–300 keV and single pulse imaging, the relativistic effects and the Coulomb force between electrons cannot be neglected [22]. In theory, a 1-D fluid model and a 2-D mean-field model are utilized to investigate the pulse duration evolution during the propagation, including the relevance with the beam spot radius [31,32].

The 1-D fluid model shows that the pulse duration is indeed affected by the beam spot radius, as shown in Appendix A. Conversely, the beam spot radius is also affected by the pulse duration. The premise of the 1-D fluid model is to assume that the beam spot radius is constant. However, the beam spot radius is constantly changing during the propagation. The 2-D mean-field model takes account of the transverse spatial distribution of the pulse and replaces the Coulomb force with an equivalent electric field, as shown in Appendix B.

### 2.2. System Layout of keV Single-Shot Ultrafast Electron Imaging

**Figure 1:**
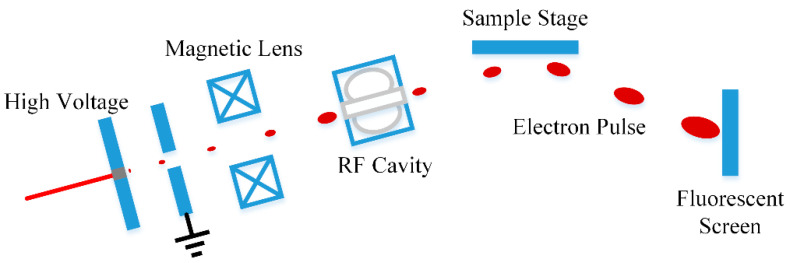
System layout of RF compression ultra-fast electron imaging.

In keV single-shot ultra-fast electron imaging systems, the initial energy dispersion and the Coulomb force during the propagation lead to inevitable expansion in longitudinal and transverse directions. Since the number of single pulse electrons is usually more than $10^{5}$, the conspicuous expansion makes it impossible to achieve an ultra-high temporal resolution. The magnetic lens focalizes the electron pulse transversely, perpendicular to the propagation direction, and the TM010 RF cavity is to compress the electrons longitudinally along the propagation direction. They are two key components to obtain high system resolution and high image quality. Based on the above theory, we proposed an ultra-fast electron diffraction system integrated into the semiconductor growth cavity for in-situ real-time monitoring of the defect formation process. The system can achieve a temporal resolution higher than 500 fs. As shown in Figure 1, ultra-fast electron pulses are excited by the femtosecond laser and then accelerated by a negative high voltage to achieve keV energy. Then the pulse is compressed by the magnetic lens and the RF cavity to realize the required resolution.

### 2.3. Simulation Conditions

The 2-D mean-field theory has been utilized to illuminate the ultra-fast electron propagation dynamics. However, our system consists of complicated processes such as deflection and compression. In addition, the Coulomb force and relativistic effects in our models cannot be neglected. Therefore, it is difficult for the revised mean-field theory to attain the ultra-high computational accuracy of femtosecond and atomic scale. Consequently, we utilize the General Particle Tracer (GPT) code to simulate the electron beam propagation dynamics during the process of excitation, acceleration, drift, transverse focus, and longitudinal compression. Based on 3-D particle-tracking techniques, the code, considering the Coulomb force between electrons and the relativistic effects, is widely utilized to investigate charged particle dynamics in electromagnetic fields. Additionally, the code, which has an embedded fifth-order Runge–Kutta driver with adaptive stepsize control, ensures accuracy during computation accelerators and beamlines [33].

Single-shot ultra-fast electron imaging requires enormous electrons in a single pulse. Thus, all simulation results below are in the magnitude of 10^5^ electrons per pulse. It has been consistently found that $2.5\times 10^{4}$ macroparticles agreed within 2% of the simulation results for $N=10^{5}$. Thus, all simulation results below used $2.5\times 10^{4}$ macroparticles here without any other approximation. The initial parameter settings are illustrated in Table 1. The initial pulse duration $\sigma_{t}$ is set to be 80 fs, multiplied by 2.35 to obtain full-width at half-maximum (FWHM) values [34]. The initial transverse root-mean-squared (RMS) radius $\sigma_{x}$ is 60 μm. The ultra-fast electron pulses are excited by a femtosecond laser incident on a metal photocathode with an energy dispersion of 0.4 eV. The initial pulse is a 3-D axisymmetric Gaussian shape in time and space. Its velocity is uniformly distributed over a spherical surface, and the velocity change within the dispersion range is uniformly distributed. The electron pulse is accelerated by 60 kV negative high voltage. The acceleration distance $d_{acc}$ is 6 mm, and the accelerating electric field strength $E_{acc}$ is 10 MV/m. The distance between the magnetic lens and the photocathode is 150 mm, and the RF cavity and the photocathode is 300 mm. The RF cavity operates in the TM010 mode interacting with electrons separated in time. Electrons at different longitudinal positions in a single pulse leave the cavity with the same temporal separation but have altered momenta [35]. These parameters are vital to our calculations. Their initial values remain unchanged unless otherwise stated.

When simulated with the GPT code, a Cartesian coordinate system was established with the center of the femtosecond laser acting on the photocathode as the origin with the pulse propagation direction as the *Z*-axis. The *X*-axis and *Y*-axis are horizontal and vertical directions perpendicular to the propagation direction, respectively. Since the cavity to be monitored is usually 400–600 mm in diameter, in the initial condition, the magnetic lens is set at z = 150 mm, the RF cavity is at z = 300 mm, the sample stage is at z = 450 mm, and the phosphor screen is at z = 600 mm. When the total current of the magnetic lens is set to 2070 A, and the compressed electric field of the RF cavity is 1.45 × 10^6^ V/m, the compressed transverse focus and the minimum pulse width can simultaneously appear at the sample stage at z = 450 mm.

## 3. Results and Discussion

### 3.1. Transverse Expansion and Longitudinal Duration

**Figure 2:**
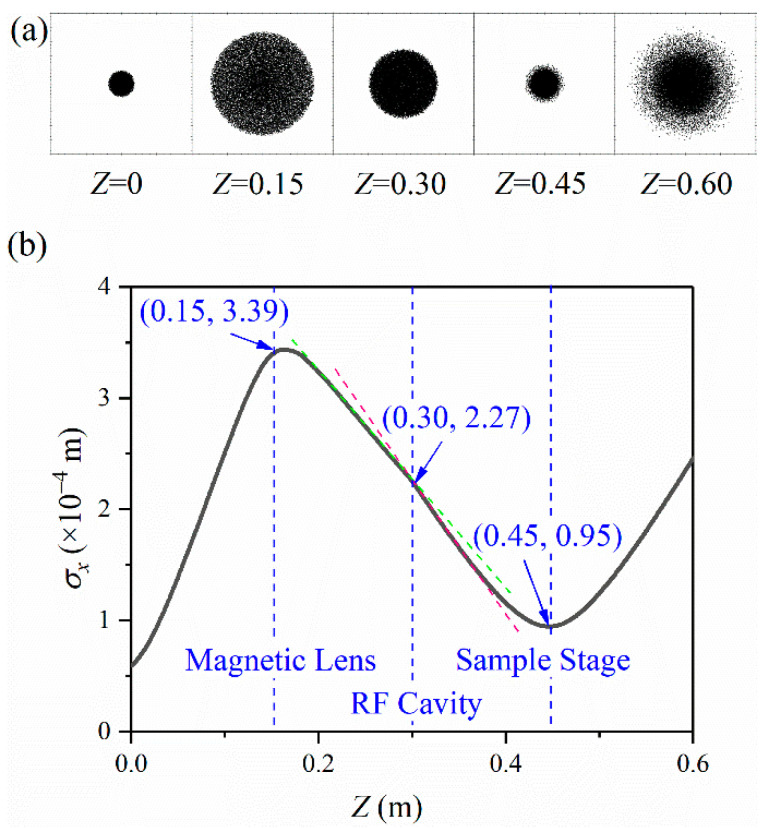
Transverse shape (**a**) and radius $\sigma_{x}$ (**b**) evolve during propagation.

Since the ultra-fast electron pulses are generated by the photoelectric effect when the femtosecond laser incident on the photocathode, the time-domain characteristics of the laser pulse are entirely copied at the moment of their generation. The energy spectrum width of the laser pulse and the scattering process of the escaping photoelectrons collectively make an energy dispersion of the electron pulses. The initial energy dispersion and the Coulomb force inevitably expand the radius and duration of the pulse [36]. The transverse shape change of the pulse during propagation is shown in Figure 2a. Since excited by the femtosecond laser on the photocathode, the pulse expands continuously. When the pulse reaches the magnetic lens at Z=0.15 m, the RMS could be up to 342 μm. Then the RMS keeps increasing. When the pulse passes through the sample stage at Z=0.45 m, the radius could reach a minimum of 95 μm. The variation of the pulse radius during propagation is shown in Figure 2b.

**Figure 3:**
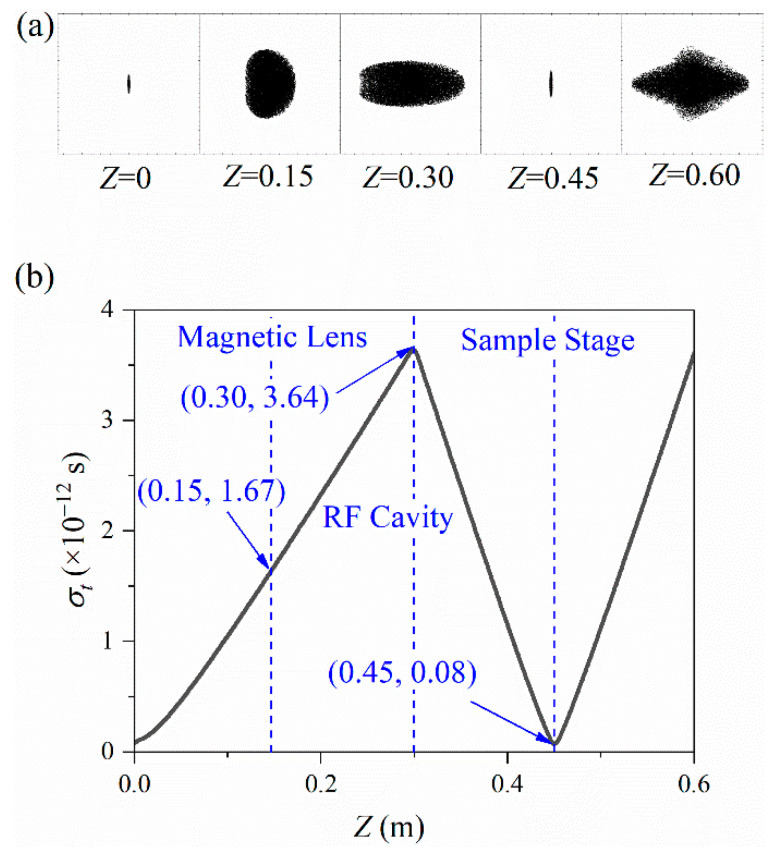
Longitudinal shape (**a**) and pulse duration $\sigma_{t}$ (**b**) evolve during propagation.

On the other hand, the pulse cross-sectional shape evolution during the propagation is shown in Figure 3a. The pulse duration evolution during propagation is shown in Figure 3b. Before reaching the RF cavity at Z=0.30 m, the pulse continues to increase to 3.6 ps. After longitudinal compression, the pulse duration decreases, reaching the minimum value of 73 fs when reaching the sample stage at Z=0.45 m. When constructing the ultra-fast electron imaging platform, we should ensure that the minimum pulse duration and focus radius are simultaneously just at the sample stage. The minimums contribute to the realization of the system’s ultra-high resolution. Our simulation results are consistent with the Luiten and Siwick group [20]. They utilized an RF cavity to compress the electron pulse duration from 3.8 ps to sub-100 fs within a distance of 620 mm and carried out experimental verification.

Combined with the analysis of Figure 2 and Figure 3, RF compression has a greater impact on the transverse focus. In contrast, the effect of the transverse compression on the longitudinal duration is negligible.

### 3.2. Effect of RF Compression on Transverse Focus

We can see that the application of the RF cavity makes the $\sigma_{x}$ curve bend, as shown in Figure 2b. This bend is also in the research results of the Luiten and Siwick group, but it has not been further explained [20]. To probe into the effects of the longitudinal compression on the radius $\sigma_{x}$, we add up to a simulation without the RF cavity. All other conditions are kept the same.

**Figure 4:**
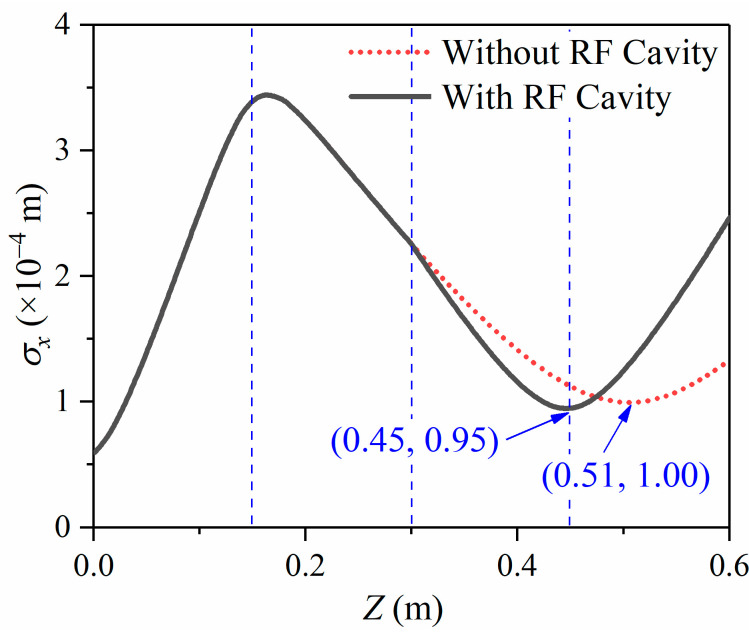
RMS radius $\sigma_{x}$ evolves during propagation with the RF cavity or not.

Figure 4 shows the variation of the pulse radius during propagation. The solid black line shows the condition with the RF cavity; the red dotted line shows the condition without the RF cavity. Except for the conditions with an RF cavity or not, the other simulation conditions are precisely the same. The RF cavity is located at Z=0.30 m. Suppose the longitudinal compression does not affect the transverse focus, in that case, the solid black line and the red dashed line should be completely coincident. However, the simulation results do not support this. The simulation results show that the red dashed line without the RF cavity is smooth at Z=0.30 m. When passing through the RF cavity, the radius curve is bent. It is generally considered that the magnetic lens and the RF cavity are mutually independent because the magnetic lens is applied to focus the electrons transversely and the RF cavity compresses the electrons longitudinally. However, the bending indicates that the RF cavity has a noticeable effect on the electron pulse’s transverse focus. The beam spot radius is reduced from 100 μm to 95 μm with a reduction of 5%. The preliminary conclusion is that the application of the RF cavity makes the transverse focus shift towards the photocathode and decreases the focus radius $\sigma_{xmin}$.

According to the theory of the 1-D fluid model and 2-D mean-field model, as shown in Appendix A and Appendix B, the evolution of pulse width, domain by time, can be obtained. This model neglects the influence of space charge effects on the lateral divergence of electron pulses. In the calculation of the pulse duration, we could obtain a better result close to the actual situation by changing the radius of the electron beam under different time steps. This model is appropriate for situations when the transverse divergence is tiny, and the longitudinal duration is apparent. In addition, the mean-field model could bring in vital errors for complex drift processes, such as acceleration and compression. Therefore, we have utilized the analysis of the X-axis velocity distribution after longitudinal compression to explain why the radius curves in Figure 4 do not coincide.

**Figure 5:**
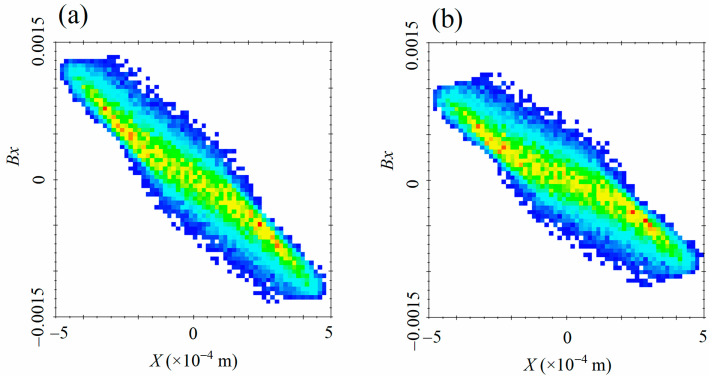
Density distribution of normalized velocity in the x-direction with the RF cavity (**a**) or not (**b**).

Figure 5a shows the distribution of the normalized velocity Bx in the *X*-axis after the RF cavity compressing. In contrast, Figure 5b shows the condition without the RF cavity. All other conditions are precisely the same. We can see from Figure 5 that the normalized velocity Bx at the end of the negative half of the *X*-axis is towards the positive direction of the *X*-axis. Moreover, the farther from the center x=0, the greater the speed is. At the same time, *Bx* at the negative half is towards the positive direction, and the further from the center, the greater the velocity is, which shows that the focusing magnetic lens is compressing the beam spot.

By contrasting Figure 5a,b, we can see that the velocity difference between the positive semi-axis and the negative semi-axis in *X*-axis with the RF cavity becomes greater than the condition without an RF cavity. The greater velocity difference leads to the beam spot radius being focused to a minimum in less time. This is because the compressed electric field of the RF cavity not only changes the longitudinal momentum but also affects the transverse momentum [32]. The conclusion is consistent with the simulation results in Figure 4.

### 3.3. Effect of Longitudinal Compression with Various Strengths and Positions on Focus Position and Focal Length

Combined with the analysis in Figure 2, the transverse focus has a negligible impact on the longitudinal compression. The pulse duration $\sigma_{t}$ curve hardly changes when electrons pass through the magnetic lens. However, longitudinal compression has a more obvious effect on the transverse focus characteristics. The radius $\sigma_{x}$ curve has a considerable bending when electrons pass through the RF cavity.

**Figure 6:**
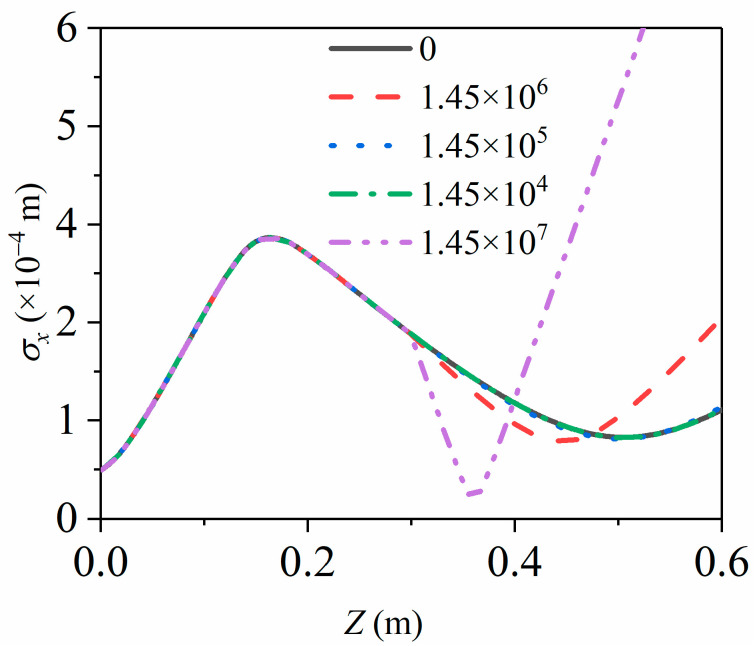
RMS radius $\sigma_{x}$ evolves during propagation at various longitudinal compression strengths, while the solid black line is 0, the red dashed line is $1.45\times 10^{6}$ V/m, the blue dot is $1.45\times 10^{5}$ V/m, the green dashed line is $1.45\times 10^{4}$ V/m and the purple double-dotted line is $1.45\times 10^{7}$ V/m.

To further probe into the effect of longitudinal compression on transverse focus, we have simulated the effect of the RF cavity on the beam spot radius under different compression strengths, as shown in Figure 6. Under the initial simulation conditions, the electron pulse obtains the minimum pulse duration and the minimum radius at 450 mm on applying the magnetic lens and the RF cavity simultaneously. Under such conditions, the compressed electric field strength of the RF cavity is $1.45\times 10^{6}$ V/m. Compared with the situation without an RF cavity compression, that is, when the compression strength is 0, three more compressed electric field gradients: $1.45\times 10^{5}\;V/m$, $1.45\times 10^{4}$ V/m, $1.45\times 10^{7}$ V/m are settled for computing the pulse duration $\sigma_{t}$ and the radius $\sigma_{x}$.

As shown in Figure 6, the pulse radius $\sigma_{x}$ curve is almost coincident at 0, $1.45\times 10^{5}$ V/m and $1.45\times 10^{4}$ V/m. When the compression strength decreases to a fixed value, the longitudinal compression has a negligible impact on the radius $\sigma_{x}$. When the electric field strength of the RF cavity increases to $1.45\times 10^{7}$ V/m, a noticeable bending appears in the radius $\sigma_{x}$ curve when pulses pass the RF cavity. The results show that the increase in the longitudinal compressive strength leads to greater effects on the radius $\sigma_{x}$. Under the current simulation conditions, the greater the intensity of the electric field in the RF cavity, the greater the impact on the momentum of the electron pulse. Existing research has shown this effect, but it has not been discussed in detail [20]. To further investigate the impact of the compression strength on the radius $\sigma_{x}$, we set more compressive electronic field gradients between $1.45\times 10^{6}$ V/m and $1.45\times 10^{7}$ V/m for analysis.

**Figure 7:**
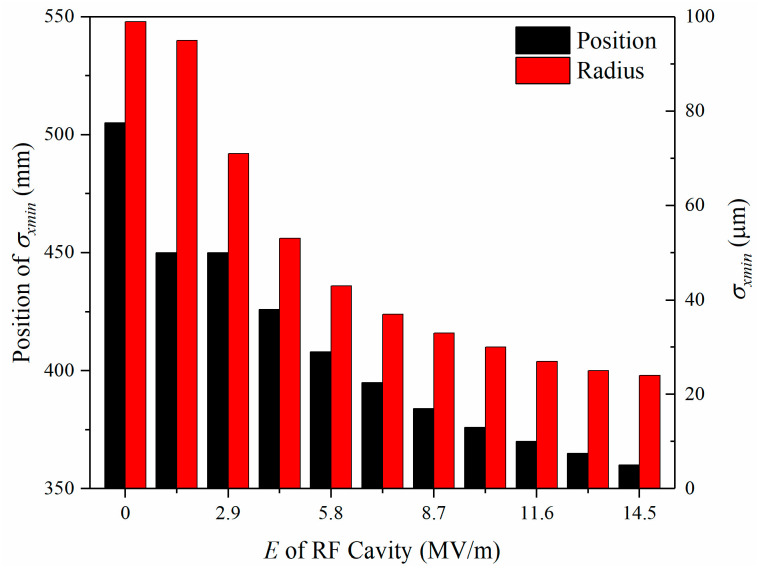
Focus radius $\sigma_{xmin}$ and its position evolve with the gradient change of the strength of the RF cavity.

When the electric field gradient for longitude compress increases from 0 to $1.45\times 10^{7}$ V/m by a multiple of $1.45\times 10^{6}$ V/m, there appears apparent changes in the focus radius $\sigma_{xmin}$, as shown in Figure 7. The focus radius $\sigma_{xmin}$ keeps decreasing, from 99 μm to 24 μm, and the corresponding position keeps shifting from 505 mm to 360 mm. The results show that increasing the longitudinal compression strength would decrease the focus radius $\sigma_{xmin}$ and shift the focus positions closer to the photocathode.

**Figure 8:**
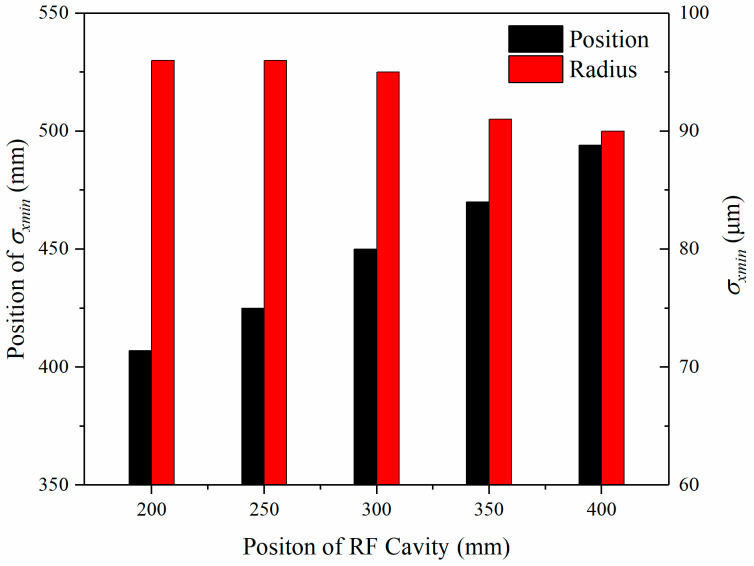
Focus radius $\sigma_{xmin}$ evolves with various positions of the RF cavity.

In addition to the impact of the longitudinal compression strength acting on the focus radius $\sigma_{xmin}$ and its position, we have simulated the focus radius $\sigma_{xmin}$ under different RF cavity positions. The change of the focus radius is not obvious, from 97 μm to 90 μm, which is negligible, as shown in Figure 8. The corresponding position evolutions are almost linear, from 407 mm, 425 mm, 450 mm, 488 mm, to 529 mm.

### 3.4. Influence on the Detection of Material Transient Mechanism

The transient evolution of materials is a highly nonlinear process, usually involving ultra-fast transitions such as free electrons and plasma concentration, photon–electron–lattice heat transfer, reflectivity, electron mobility, and other photoelectric characteristics [37,38]. The detection of the transient nonlinear process (heat transfer between electrons (fs), heat transfer between the hot electrons and the lattice (ps), and heat transfer between the lattice (ns)) needs an ultra-high temporal resolution [39]. The detection needs an ultra-high spatial resolution because people wish to observe the microscopic evolutions of electrons and lattice.

Combined with the results in this paper, the longitudinal duration is on the order of femtoseconds, and the beam spot radius is on the order of hundreds of microns for ultra-fast electron beams. The spatial resolution of electron diffraction can theoretically reach the order of Angstroms ($10^{-10}$ m) according to the Rayleigh criterion [40]. It is challenging to extract the characteristic signal of the lattice because the beam spot radius is usually with a radius of hundreds of microns.

The study in this paper shows that the RF cavity is a critical component for longitudinal compression. Moreover, its influence on the transverse focus cannot be ignored for the ultra-fast electron beam. The beam spot radius can be reduced from 100 μm to 30 μm on the action of the RF cavity under careful adjustment. The reduction in the radius can reduce the area of extracting weak signals. Furthermore, under the premise that the number of electrons in a single pulse remains constant, the smaller the radius, the greater the number of electrons per unit area acting on the sample, and the detected signal is more robust. Therefore, in the detection of a material transient mechanism, longitudinal compression is not only beneficial to improve the temporal resolution but also beneficial to the extraction of weak signals. This conclusion will change people’s habitual opinion that longitudinal compression will only improve the temporal resolution of the system [26].

## 4. Conclusions

The propagation process of ultra-fast electron beam for detecting the transient evolution of materials was studied by solving the fifth-order Runge–Kutta equation based on the 1-D fluid model and the 2-D mean-field model. We emphatically simulated the influence of RF compression under various intensities and positions on the transverse focus. Our study changes the opinion that the RF cavity only has the function of compressing the longitudinal pulse duration [32] and complements the theory of the effects of the RF cavity on the transverse focus power [26]. The results show that the RF cavity is not only a critical component for longitudinal compression but also influences the transverse focusing. In contrast, the effect of the transverse compression on the longitudinal duration could be negligible. The application of the RF cavity makes the transverse focus shift towards the photocathode and decreases the focus radius. When the electric field strength of the longitudinal compress increases from 0 to $1.45\times 10^{7}$ V/m by a multiple of $1.45\times 10^{6}$ V/m, the focus radius keeps decreasing, from 99 μm to 24 μm, and the corresponding position shifts from 505 mm to 360 mm. However, in different RF cavity positions, the focus radius evolution is negligible, from 97 μm to 90 μm. The corresponding position evolutions are almost linear from 407 mm, 425 mm, 450 mm, 488 mm, to 529 mm. The beam spot radius can be reduced from 100 μm to 30 μm on the action of the RF cavity under careful adjustment. The electrons density incident on the sample could be increased from $3.18\times 10^{12}$ $m^{-2}$ to $3.54\times 10^{13}$ $m^{-2}$ when the number of single pulse electrons remains constant. The larger the electron density incident on the sample, the greater the signal intensity, which is more conducive to detecting the transient evolution of the material.

## Figures and Tables

**Table 1:** Main parameters and initial values.

Main Parameters	Initial Values
Electron number per pulse N	$10^{5}$
Pulse duration $\sigma_{t}$ (RMS)	80 fs
Transverse radius $\sigma_{x}$ (RMS)	60 μm
Energy dispersion $E_{0}$	0.4 eV
Acceleration electric field $E_{acc}$	10 MV/m
Acceleration distance $d_{acc}$	6 mm

## Data Availability

Some or all data, models, or code that support the findings of our study are available from the corresponding author upon reasonable request.

## Nomenclature

XRD	X-ray diffraction
RF	radio frequency
GPT	general particle tracer
FWHM	full width at half maximum
RMS	root mean squared
N	electron number per pulse
$\sigma_{t}$	pulse duration
$\sigma_{x}$	transverse radius
$E_{0}$	energy dispersion
$E_{acc}$	acceleration electric field
$d_{acc}$	acceleration distance
Bx	normalized velocity in X-axis

## Appendix A

In the 1-D fluid model, the radius is assumed to be constant, and the pulse duration $\Delta t_{fluid}$ is given by [32]:

(A1) $$\Delta t_{fluid}=\Delta t_{0}+\frac{Ne^{2}}{2m\varepsilon_{0}\nu r_{e}{}^{2}}t^{2}$$

where $\Delta t_{0}$ is the initial pulse duration, N is the number of electrons contained in the electron pulse, ν is the velocity of the electron pulse, $r_{e}$ is the radius of the electron beam, t is the propagation time of the electron pulse, $\varepsilon_{0}$ is the permittivity of free space, e, m, and ν are the charge, mass, and velocity of the electrons, respectively.

## Appendix B

The differential equation of the 2-D mean-field model is given by [32]:

(A2) $$\frac{dl^{2}}{dt^{2}}=\frac{1}{\kappa }\cdot \frac{Ne^{2}}{m\varepsilon_{0}\pi r_{e}{}^{2}}\left(1-\frac{l}{\sqrt{l^{2}-4r_{e}{}^{2}}}\right)$$

where l is the spatial length of the electron pulse on the Z-axis, κ is a parameter related to the shape of the electron pulse. κ~1 for uniform distribution and κ~2 for Gaussian distribution.
